# Research on Forward Problem of Rail Detection Based on Magnetoacoustic Coupling

**DOI:** 10.3390/s22155539

**Published:** 2022-07-25

**Authors:** Xin Huang, Aijuan Li, Zhen Huang, Yi Sun, Yumin Song, Ning Xu

**Affiliations:** 1 School of Electrical Engineering, Shan Dong Jiaotong University, Jinan 250357, China; 205045@sdjtu.edu.cn; 2 School of Automotive Engineering, Shan Dong Jiaotong University, Jinan 250357, China; syumin@126.com; 3 SWAT Corps, Shandong Provincial Public Security Department, Jinan 250115, China; hz18615173617@163.com; 4 Field Centre, Shandong Provincial Agricultural Machinery Scientific Research Institute, Jinan 252100, China; 13791066727@163.com

**Keywords:** magnetic acoustic imaging, pulse current, magneto-acoustic signal, rail crack

## Abstract

According to the characteristics of rail defects, a rail microcrack detection method based on magnetoacoustic coupling effect is proposed in this paper. Firstly, the basic principle of a rail microcrack detection method based on magnetoacoustic coupling effect is described, and then the model is analyzed theoretically. Through simulation calculation, the current density distribution and Lorentz force distribution generated by electromagnetic excitation, the motion characteristics of particles under Lorentz force and the sound field distribution characteristics of magnetoacoustic signals generated by Lorentz force are obtained. Finally, an experimental platform was set up and the steel ring model was preliminarily tested. The magnetic and acoustic signals of the two steel ring boundaries excited by an electromagnetic field were collected. These signals correspond to the position distribution of the steel ring. The state change of rail microstructure will cause a change in the conductivity characteristics of rail materials, and will affect the characteristics and distribution of sound pressure in the detection. Therefore, the detection method based on the magnetoacoustic coupling effect can detect the surface microcracks of high-speed rail. This method has great feasibility and development potential in the field of rail flaw detection.

## 1. Introduction

Rail transport is the most efficient and relatively inexpensive way to transport passengers and goods over long distances. The rapid development of railway has not only changed people’s lifestyles, but has also had a far-reaching influence on world politics and the economy [1]. The US rail network is the largest in the world, operating more than 250,000 km of routes, with freight lines accounting for about 80 per cent of the country’s total network. By the end of 2020, China had 146,000 km of railway operating miles, an increase of 5.3% over the previous year, including 38,000 km of high-speed rail. The rate of double-track railway is 59.5%, and the rate of electrification is 72.8%. By the end of 2020, China had 146,000 km of railways in operation, up 5.3% from the end of 2019, including 38,000 km of high-speed railways. The railway multitrack rate was 59.5%, and the electrification rate was 72.8%. The density of China’s railway network was 152.3 km/10,000 km^2^, 6.8 km/10,000 km^2^ more than in 2019. In 2020, 2.203 billion passengers traveled, down 39.8% from 2019. The total volume of goods delivered by rail was 4552 billion tons, an increase of 3.2% over the previous year. In the same year, the total turnover of goods reached 3051.446 billion tons and kilometers, an increase of 1.0% [2]. Railway transportation plays an irreplaceable role in China’s transportation industry [3]. The huge carrying capacity has brought a severe test to the health of the rail network. With the implementation of the railway speed-up strategy, the traffic density of the train has increased, which has further increased the burden on the rail network, and also increases the macro damage risk to the rails, such as bending, torsion, compression, elongation, wear and fracture, which pose a major threat to the safe operation of the train [3,4]. The largest proportion of these damages is the contact fatigue damage caused by long-term stress accumulation [5], which is the main hidden danger threatening the safe operation of the rails [6]. This kind of damage exists in the form of small cracks at the initial stage. With the accumulation of plastic deformation of the rails, the fine cracks will further expand. Fine cracks can lead to rail fracture and block falling [7]. These problems can cause the rail to break, resulting in fatal accidents such as car destruction and human deaths [8,9]. Therefore, rapid and effective detection of rail microcracks is very important to ensure the safety of railway operations.

With the rapid development of railways, the requirement of rail NDT increases. The types of crack damage are complex and varied, and the propagation modes of crack damage are changing constantly, which put forward higher requirements for rail nondestructive testing technology [10]. The existing detection methods are not only single, but also have a huge gap with the actual field application [11]. Since the world’s first rail ultrasonic testing vehicle was put into use in 1959, non-destructive testing technology has been widely used in rail crack detection and patrol inspection. In the crack detection of high-speed rail, the railway rail nondestructive testing technologies studied and applied at home and abroad mainly include ultrasonic testing, electromagnetic testing, thermal imaging testing and visual testing [12,13,14,15]. Among them, the ultrasonic testing method is widely used in rail flaw detection in China [16]. Ultrasonic technology has strong penetration ability and is suitable for automatic scanning detection, but ultrasonic technology has a surface detection blind area, which makes it difficult to detect surface and near-surface fatigue crack damage within 4mm below the material surface [17]. However, different detection methods correspond to different instruments, and the detection principles and performance of instruments are different. These methods and instruments have some limitations. Conventional nondestructive testing methods such as ultrasound can only detect internal defects of a certain size that have developed, and it is difficult to carry out effective evaluation of early damage in rails. In the stage of defect formation and propagation, namely the fatigue process, it is necessary to observe the microstructure of the material for early damage detection and rail failure early warning. During the fatigue process, that is, the process of producing defects, electrical conductivity changes synchronously. The magneto-acoustic coupling detection technology can detect the early damage of rail by detecting the change in rail conductivity, and then realize the early warning of rail fault.

Electromagnetic acoustic imaging (mmtai) is a nondestructive testing and evaluation method based on object characteristics developed in recent years. The magneto-acoustic coupling detection technology obtains the ultrasonic signal by electromagnetic excitation, and then uses the ultrasonic signal to reconstruct the electrical conductivity distribution image of the object. In 2014, the Institute of Electrical Engineering, Chinese Academy of Sciences, systematically studied mmtai imaging and obtained the magnetoacoustic signal of low-conductivity medium under low-frequency magnetic fields. At the same time, the group studied the injection current magnetic acoustic imaging technology and realized the low conductivity tissue imaging with a resolution of 0.8 mm [18]. The Chongqing University examined the magnetic-acoustic effects of metal materials, demonstrating the possibility of magnetic-acoustic imaging [19].

In this paper, the multi-physical field coupling method is used to realize real-time detection of rail crack. The multi-physical field method includes electric fields, magnetic fields and sound fields. By developing high-resolution and high-contrast imaging technology, crack imaging on the surface and inside of rail can be realized. This method has the following advantages:

(1) As the excitation source, the pulse electrical signal can directly excite the magnetoacoustic signal in the rail, and the generated signal is easily received by the sensor, so it can detect in various harsh environments; (2) the control of the pulse electric signal source is very flexible and can be easily moved near the defect near field, which provides convenience for rail defect detection; (3) through the adjustable magnetic field space and the focusing detection of the magnetoacoustic signal receiver, the ultrasonic detection resolution can be realized.

Further, this detection method combines magnetic acoustic detection with spectrum analysis. Magnetoacoustic signals are characterized by low frequency and narrow band width. In theory, this detection method can analyze the mechanical information of the rail with the magneto-acoustic signal, because the information is related to the defects of the rail, so this detection method can obtain a higher resolution image than the frequency of the magneto-acoustic signal receiver. Magnetic acoustic detection has high practical value in rail inspection. It is a new non-destructive inspection technology with great application background.

## 2. Theoretical Basis of Magneto-Acoustic Coupling Detection

**Figure 1:**
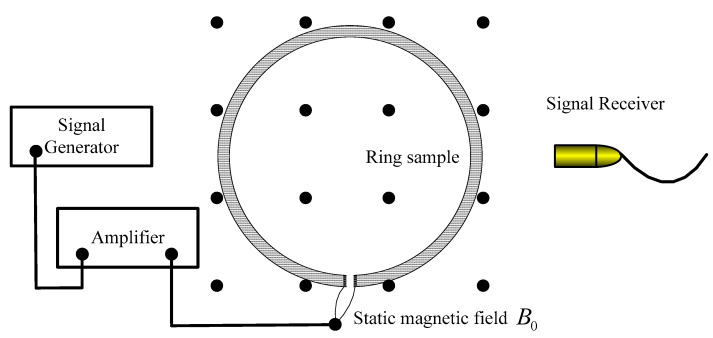
Magnetoacoustic coupling effect.

Magnetoacoustic coupling imaging detection technology is a typical electromagnetic-ultrasonic imaging method [20,21]. Magnetoacoustic coupling imaging detection technology is divided into induction magnetoacoustic coupling imaging detection technology and injection current magnetoacoustic coupling imaging detection technology. Firstly, this paper studies the injection current magnetoacoustic coupling imaging detection technology [22,23,24,25]. The magnetoacoustic signal is generated by the magnetoacoustic effect. The principle is shown in Figure 1. In Figure 1, the black spot distribution region represents the magnetic field region.

The magnetoacoustic effect is that when the alternating current flows through the object in the static magnetic field, the current generates Lorentz force under the action of the static magnetic field, resulting in the rise of local pressure, which is then transmitted in the form of pressure wave to form the phenomenon of ultrasonic wave. The generation of magnetoacoustic signals through the magnetoacoustic effect needs to go through two physical processes: the process of Lorentz force sound source generated by the interaction of time-varying current and static magnetic field, that is, the forward problem of electromagnetic field; and the process of transmitting the sound field generated by the sound source to the outside, that is, the sound field forward problem. In order to realize the above two processes, microsecond electromagnetic pulse is selected to excite magnetoacoustic signals [26].

In this paper, the forward problem of electromagnetic field is discussed, that is, the process of producing Lorentz force source by the interaction of variable current and a static magnetic field. The model is built according to the actual test equipment and model parameters, and the actual experimental environment is simulated according to the actual test equipment and model parameters. During the simulation, the steel ring was chosen as the experimental model. Four aspects are calculated in the simulation process: first, the corresponding relationship between the current density distribution and the exciting current in the steel ring, and second, the corresponding relationship between the Lorentz force and the current density. In the next step, the motion characteristics of the particle in the steel ring under the action of Lorentz force were described. Finally, the acoustic field characteristics of the original sound pressure were calculated in 3D. The simulation results can provide theoretical guidance for the actual experimental detection.

Taking the rail model as an example, the forward problem of electromagnetic field is described. The magnetic vector potential component ϕ in the model can be obtained by Equation (1).

(1) $$\left\{ \begin{array}{l} \nabla^{2}A-\mu \sigma \frac{\partial A}{\partial t}=-\mu J_{s}\;\;\;\\ {n\times \left(\frac{1}{\mu }\nabla \times A\right)|}_{\Gamma_{1}}=0\;\;\;\;\;\;\;\; \end{array}\right.$$

where, A is the component in the direction ϕ of the magnetic vector potential in the model. The initial stress field ***f*** generated by Lorentz force can be calculated by Equation (2).

(2) $$f=J\times B_{0}=\sigma E\times B_{0}=-\frac{\partial A}{\partial t}B_{0}e_{r}\;$$

Lorentz force ***f*** is the source term of solid vibration. According to the principle of continuum mechanics [27,28], in an inertial reference system, the vector form of the Navier Stokes equation of elastic solid is Equation (3).

(3) $$\{\begin{array}{c} \rho \frac{\partial^{2}u}{\partial t^{2}}=G\nabla^{2}u+\frac{G}{1-2\nu }\nabla \left(\nabla \cdot u\right)+f\;\mathrm{in}{\;\Omega }_{1}\\ \;f=-n_{s}p\;\mathrm{in}\;\Gamma_{1} \end{array}$$

In the formula, $u=u\left(x_{1},x_{2},x_{3},t\right)\;$ is the displacement field, ρ is the density of the model, G is the shear modulus, ν is the Poisson’s ratio, f is Physical Strength, which is the Lorentz force of the model in the injected current magnetic acoustic imaging. and $n_{s}$ is the unit normal vector pointing outwards on the boundary. The solution equation of sound field is Equation (4).

(4) $$\{\begin{array}{c} \nabla^{2}p-\frac{1}{c_{s}^{2}}\frac{\partial^{2}p}{\partial t^{2}}=0\;\mathrm{in}\;\Omega_{2}\\ \;p=0\;\;\mathrm{in}\;\Gamma_{2}\\ n\cdot \nabla p=n\cdot \frac{\partial^{2}u}{\partial t^{2}}\;\mathrm{in}{\;\Gamma }_{1}\\ \;r=0\;\;\mathrm{on}\;\mathrm{axles} \end{array}\;$$

Through the above description, it can be found that the initial stress field is determined by electromagnetism, mechanics and conductivity parameters. However, f is obviously affected by material properties. When subjected to electromagnetic excitation, the steel body in a certain depth under the surface will produce an initial sound field. When the rail has defects, due to different material characteristics, the Lorentz force is different from that of the rail without defects, and different defects will produce different Lorentz forces. Lorentz force is the source term of solid vibration. It propagates outward in the form of sound field, that is, magnetoacoustic signal. By collecting the magnetoacoustic signal and reconstructing the algorithm, the sound pressure distribution map of different defective rails can be drawn, so as to achieve the purpose of observing and identifying rail defects. In this paper, the Lorentz force sound source generated by current under the action of static magnetic field is simulated by the axisymmetric model.

## 3. Simulation Calculation

In order to verify the effectiveness of the method, taking the axisymmetric model steel ring as an example, the finite element software is used to simulate the generation process of the magnetoacoustic effect.

**Figure 2:**
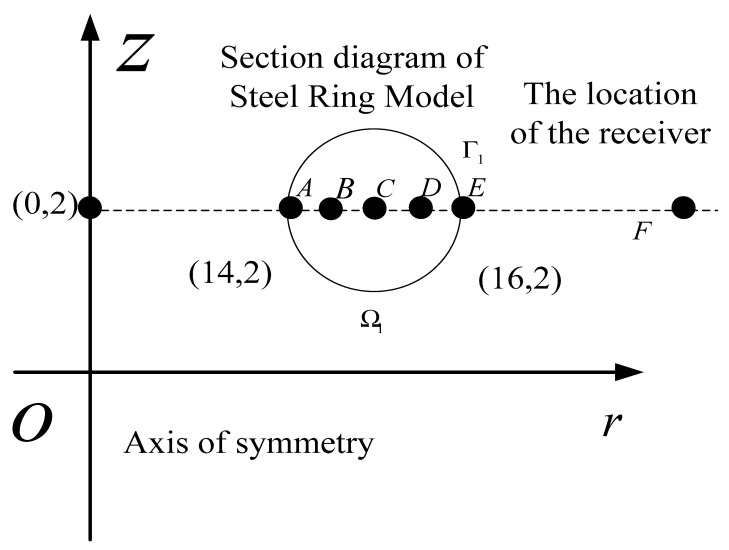
Schematic diagram of the steel ring model.

The steel ring is selected as the experimental model, the inner radius is 14 mm, the outer radius is 16 mm, the cross-section area is $\pi \times 1{\;\mathrm{mm}}^{2}$, the ring is placed in the horizontal plane, and its central axis is along the vertical direction. The direction of the static magnetic field also follows the vertical direction and is evenly distributed in the space where the model is located. The magnetic flux density of the static magnetic field is $B_{0}$, as shown in Figure 2. The signal generator outputs the pulse signal, which is amplified by the amplifier and loaded on the steel ring. The original sound field excited by the steel ring is received by the magnetoacoustic signal receiver placed around the steel ring. The steel ring is an axisymmetric model, so the research only needs to study the characteristics of the electromagnetic field and physical field on its meridian plane.

**Figure 3:**
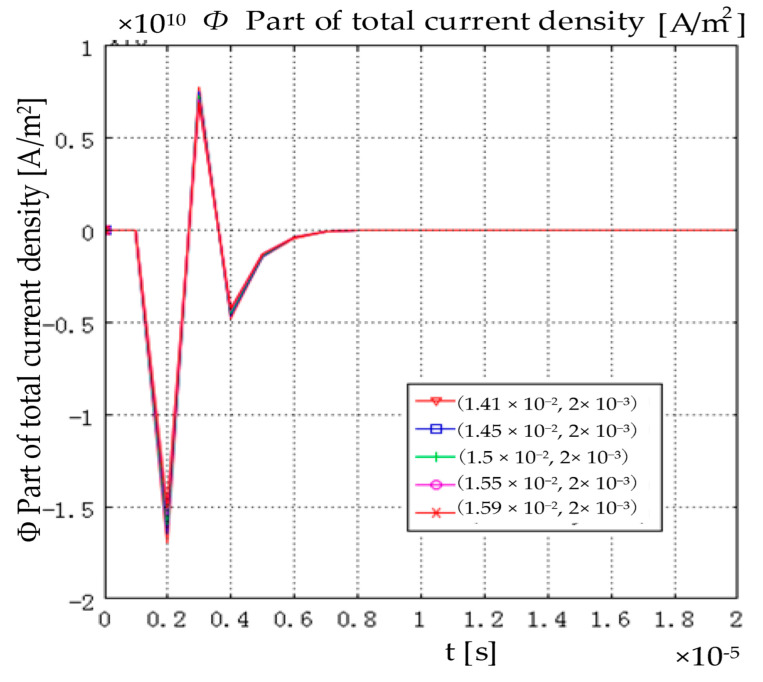
Part of the total current density at each point in the model.

**Figure 4:**
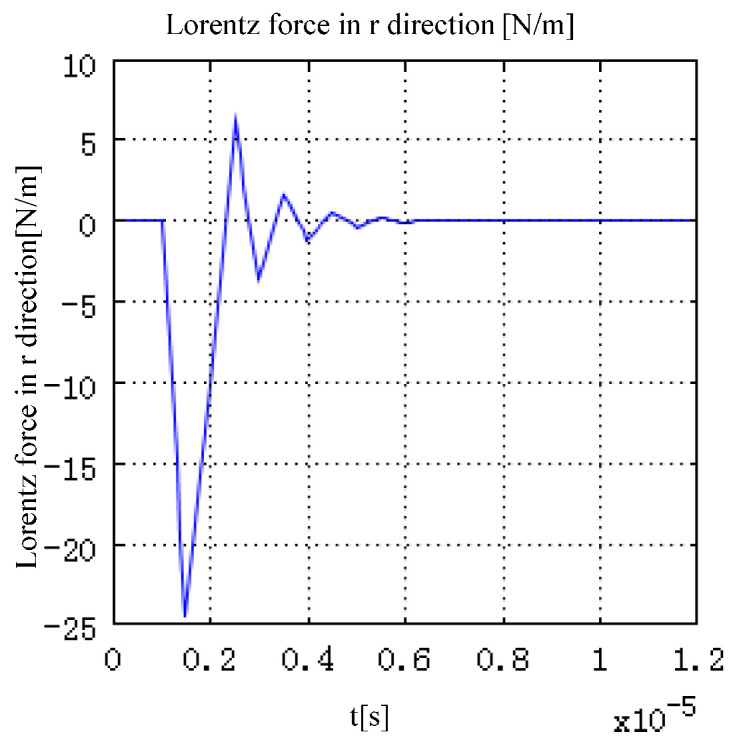
Lorentz force in direction.

**Figure 5:**
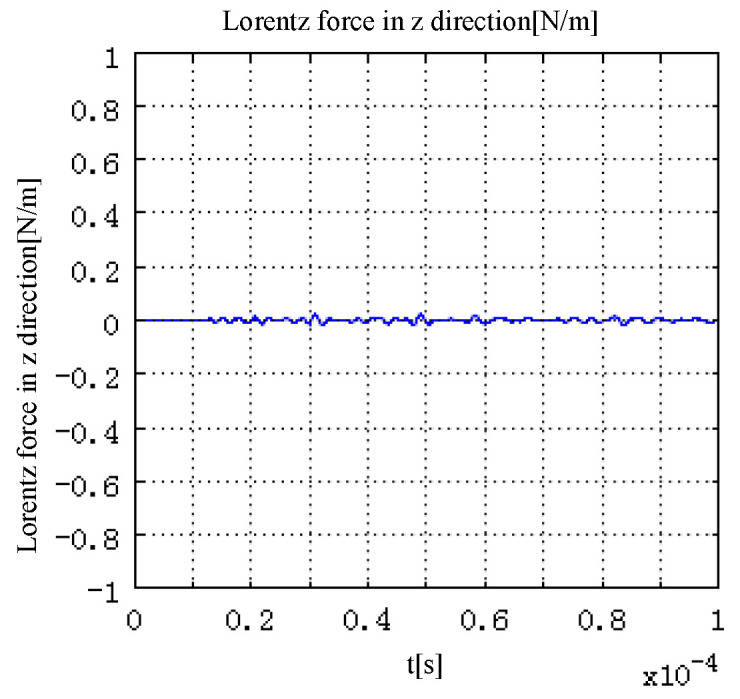
Lorentz force in z direction.

**Figure 6:**
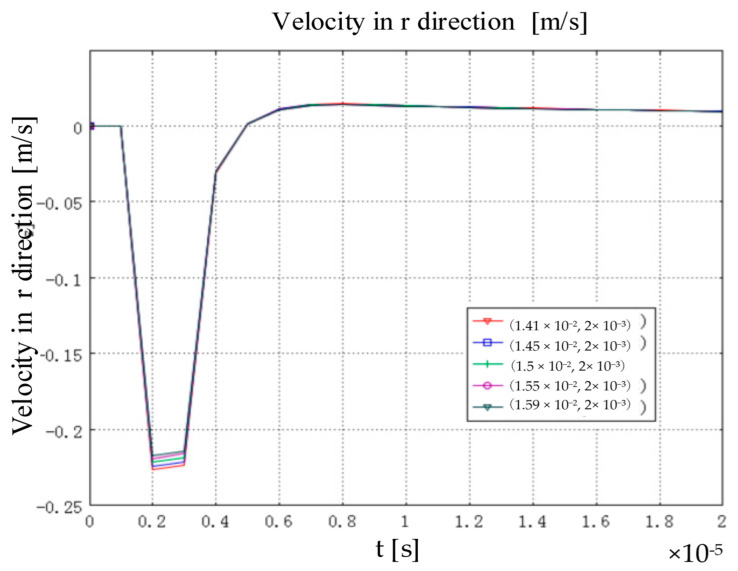
Velocity in r direction [m/s].

**Figure 7:**
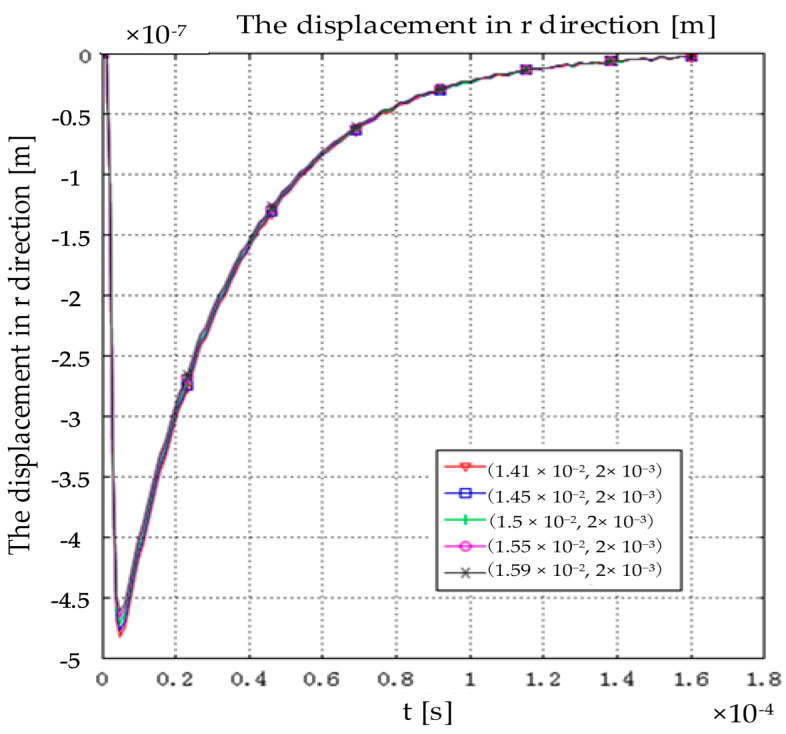
The displacement in r direction [m].

As shown in Figure 2, in coordinate system r−o−z, the coordinate of the central point of the steel ring is (0,2 mm) and the coordinate of the center of its meridian plane is (15 mm,2 mm). The solution of the multi physical field problem is divided into two areas: the electromagnetic field solution area covers the model $\Omega_{1}$ to be imaged to obtain the electromagnetic field information in the model; in the solid vibration solution area, only the model $\Omega_{1}\;$ to be imaged is solved to obtain the particle displacement. Three problems are analyzed in the simulation analysis; the first is the vibration of internal particles in the steel ring model. The second is the vibration characteristics of the sound source, and the third is the distribution characteristics of the sound source. Therefore, five inspection points on the circular section of the steel ring are selected from the inside to the outside:  A(14.1 mm, 2 mm), B(14.5 mm,2 mm), C(15 mm,2 mm), D(15.5 mm,2 mm), E(15.9 mm,2 mm), as shown in Figure 2. In Figure 3, Figure 4, Figure 5, Figure 6 and Figure 7, the electromagnetic field data and particle vibration data detected at these five different positions are represented by “∇, □, +, ⊙, ×“ icons, respectively.

### 3.1. Electromagnetic Characteristics of Each Point in the Model

When a current signal with a pulse width of 1 μs is injected into the two-dimensional axisymmetric model, the total current density distribution of each point in the model is shown in Figure 3. Under the action of the rising edge of the applied current signal, the current density in the model first generates a pulse in the opposite direction. Due to the narrow pulse width of the excitation electric signal, under the action of the falling edge of the applied current signal, the current density waveform in the model then generates a forward pulse. Then, it is returned to 0 after vibration; the amplitude difference of the current density waveform of each point in the model is very small. It can be considered that the change in the current density of each point in the model with time is the same. That is, in the injection current magnetoacoustic imaging, the change of the current density of each point in the model with time is synchronous.

The values of Lorentz force generated by electromagnetic field interaction in directions r and *z* are shown in Figure 4 and Figure 5. The time characteristic of the excitation pulse determines the time characteristic of the Lorentz force generated in the model. As shown in Figure 4, with the change in the current density in the model, the Lorentz force in direction r in the model first increases along the negative direction of r. After reaching the peak value, the peak value gradually decreases, and then increases along the positive direction of r. Finally, it returns to 0. The change in the 1 direction of the Lorentz force corresponds to the change in the total current density in the model.

As is shown in Figure 5, since the magnetic field is along the direction z, the Lorentz force in the direction z in the model is far less than that in the direction r, which is almost negligible. It can also be seen that when the pulse width of the excitation current is 1 μs, the pulse width of the Lorentz force waveform in the direction inside the model is 2 μs. When the excitation current density is Jδ(t), the Lorentz force in the model is approximately a function of fδ(t).

### 3.2. Sound Source Characteristics in Steel Ring

The particles in the rail will produce vibration and displacement under the action of Lorentz force. The following are the time characteristics of vibration velocity and displacement of particles in the model in different directions. Figure 6 shows the time characteristics of particle vibration velocity along the direction of particle r in the steel ring. The vibration velocity difference of particles along the r direction at the five groups of detection points is very small. The maximum vibration velocity of the particle along the r direction is 0.23 m/s. In the process of one vibration, the vibration velocity of particle increases first, then decreases along r, and finally returns to zero.

Figure 7 shows the r direction displacement of the particle under the action of Lorentz force. Along r directions, the particle first reaches the extreme value in r directions, then returns to the origin; the whole process does not oscillate, and the maximum displacement of the particle in r directions is $-4.8\times 10^{-7}\;m$.

The results show that there is a strong correlation between the current density and the vibration of particles in solids. According to the distribution of sound sources, the component of vibration velocity and displacement along r direction is large. Therefore, the ultrasonic signal excited by the model in r direction is strong. The vibration velocity and displacement of particles in directions are much less than those in *r* directions. In future experiments, this conclusion can be used to select the receiver when the location of the magnetic acoustic signal is determined.

### 3.3. Waveform of the Initial Sound Field

**Figure 8:**
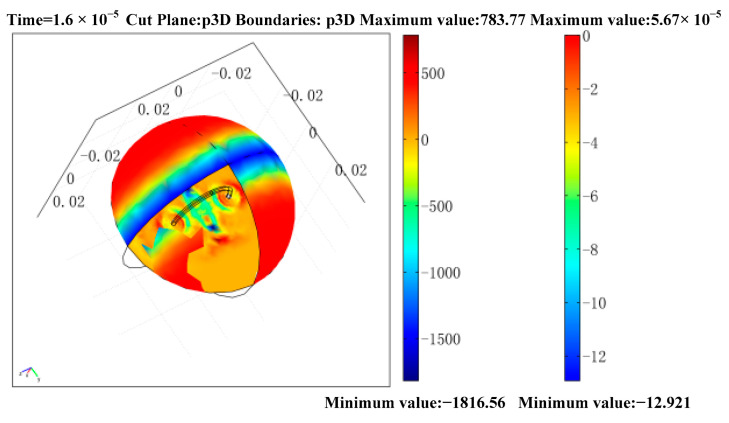
Distribution of sound pressure on the section and boundary of the 3-d model.

The sound field propagates through the whole background area $\Omega_{1}$, and the two-dimensional axisymmetric model has only one cross-section. This two-dimensional cross-section cannot reflect the change of the whole space sound field. It is necessary to draw a three-dimensional model graph to study the change of sound field comprehensively. The calculation of three-dimensional model is very heavy. In order to reduce the computational effort, the results can be skimmed using the stretch coupling variable in the Comsol physics software, whose function is to transform the variables or expressions in a model according to a custom coordinate, directly into another model. In the sound field analysis, firstly, the sound field propagation simulation under a two-dimensional axisymmetric model is established, and the distribution of the sound field is obtained after calculation. At this point the variable only exists in the two-dimensional axisymmetric model. Then, a new three-dimensional geometry is built under the same model. The three-dimensional geometry is generated by rotating the two-dimensional axisymmetric geometry directly around the axis of symmetry. Using the stretching coupling variable, the variable P in the 2-d axisymmetric model is transferred, and then the variable  3D, 3D in the 3-d geometry are detected as the sound pressure in the 3-d model. By rotating the two-dimensional axisymmetric geometry, the three-dimensional geometry is obtained as shown in Figure 8. The middle ring is a steel ring sample, and the surrounding spherical area is the sound field propagation area.

**Figure 9:**
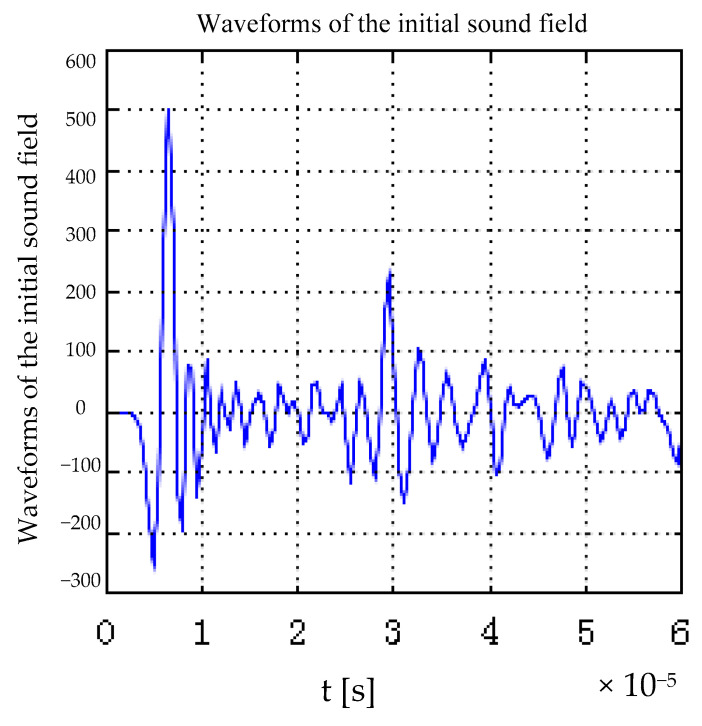
Waveform of the initial sound field.

After the original sound field is excited, the original sound field propagates outwards. In the course of the propagation of the original sound field, the amplitude of the sound pressure will change due to the attenuation and other reasons. Figure 9 is the original sound field waveform detected by the magneto-acoustic receiver at position F(0, 2 mm).

As can be seen from Figure 9, two acoustic signals are detected at the detection point. The first acoustic signal is excited from the steel ring boundary near the detection point. The second acoustic signal is excited by the boundary far from the detection point, the amplitude of the second ultrasonic signal is less than the amplitude of the first ultrasonic signal. That is, the farther away the detection point is from the sound source, the smaller the amplitude of sound pressure transmitted by the sound source.

### 3.4. Simulation Calculation of Magneto-Acoustic Signals with Different Models

**Figure 10:**
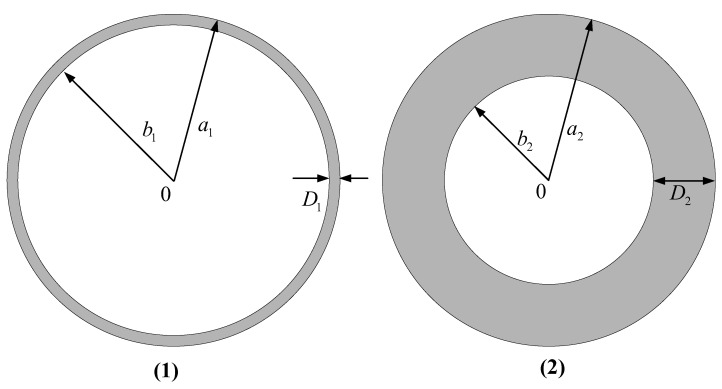
Magnetoacoustic signal simulation model.

The ring model and the disc model as shown in Figure 10 are built to simulate the magnetoacoustic signal. (1) is ring model and (2) is the disk model. The outer radius of the ring model is $a_{1}=15\;\mathrm{mm}$, and the inner radius is $\;b_{1}=14.9\;\mathrm{mm}$; The outer radius of the disc model is $a_{2}=15\;\mathrm{mm}$, and the inner radius is $b_{2}=9\;\mathrm{mm}$; The sampling radius of the signal is $r_{d}=100\;\mathrm{mm}$.

**Figure 11:**
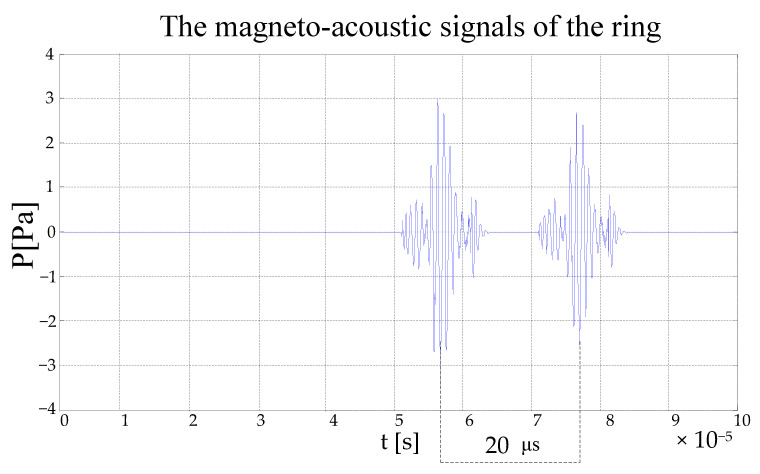
The magneto-acoustic signal waveform of the ring.

The electromagnetic excitation is applied to the ring model, and the ring generates a magneto-acoustic signal through an electromagnetic coupling effect. When the scanning radius is, the magneto-acoustic signal acquired waveform as shown in Figure 11.

As shown in Figure 11, the ring is excited to produce two sound waves. The time interval between two sound waves is, The time interval corresponds to the time of the propagation of the ultrasound between the two boundaries of the ring. Therefore, these two sound waves are the sound signals excited by the two boundaries of the ring.

**Figure 12:**
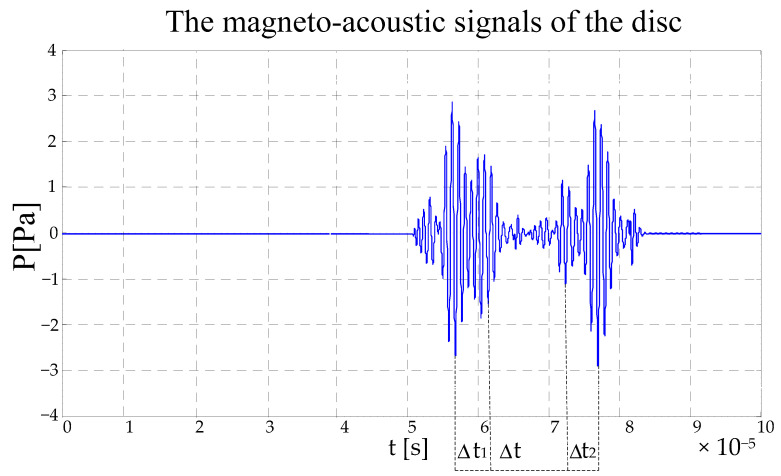
The magnetoacoustic signal waveform of the disc.

The electromagnetic excitation is applied to the disc model, and the disc generates a magnetoacoustic signal through an electromagnetic coupling effect. When the scanning radius is, the magneto-acoustic signal acquired waveform as shown in Figure 12.

In Figure 12, there are two sound waves. The relative times of the pulses in the sound waves are calculated; from the calculation results, it can be seen that the position of sound pulse is corresponding to the position of the ring boundary. The width of the sound wave string corresponds to the propagation time of the sound wave in the disc. In Figure 12, there is no sound wave in the corresponding part of time, which corresponds to the propagation time of the sound wave in the middle part of the disc. Because of the close distance between the inner and outer boundaries of the disc, the magnetoacoustic signals are superimposed, which is determined by the shock function of the ultrasonic transducer system.

## 4. Experimental Results and Analysis

**Figure 13:**
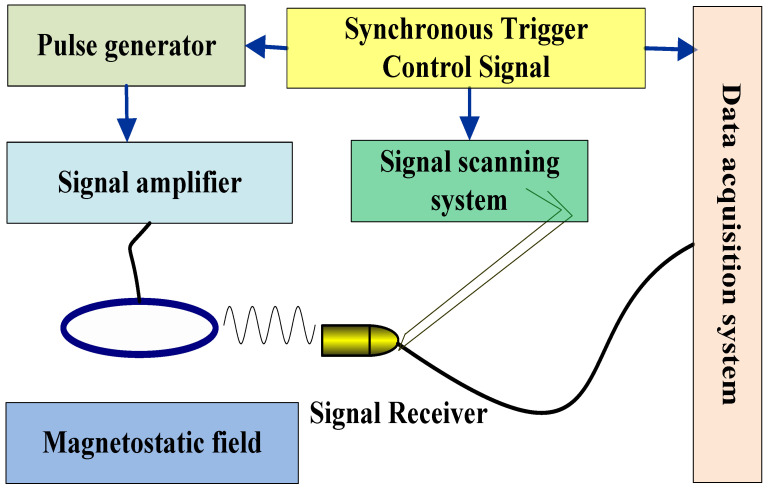
Experimental platform.

**Figure 14:**
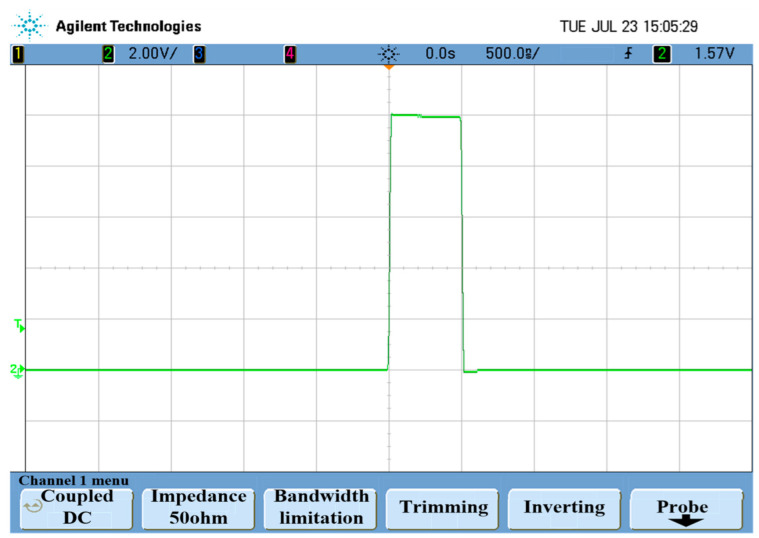
Exciting signal waveform in experiment.

In order to further verify the rationality and feasibility of the method, a high-speed rail defect magnetic acoustic detection platform is designed, the structure of which is shown in Figure 13. The system includes signal excitation system, signal receiving system and acquisition system. The pulse generator sends out a Pulse Electric Signal, which is amplified by the amplifier and loaded on the steel ring. The signal scanning system is synchronously triggered to receive the magnetic sound signal, the received magneto-acoustic signal is collected and processed after passing through the signal detection circuit. The excitation signal is a pulse wave with the waveform shown in Figure 14.

The experimental model is a steel ring, the diameter of the steel ring is m, and the diameter of the steel ring is D=30 mm. In the experiment, the scanning radius of the magnetoacoustic signal receiver is r and the diameter of the detection plane is 15 mm. The sampling frequency of the oscilloscope is 1 GHz. The pulse square wave signal had a signal generator output frequency of 10 kHz and a duty cycle of 0.5%. As shown in Figure 11, the ring is loaded after being amplified 20 times by a power amplifier.

**Figure 15:**
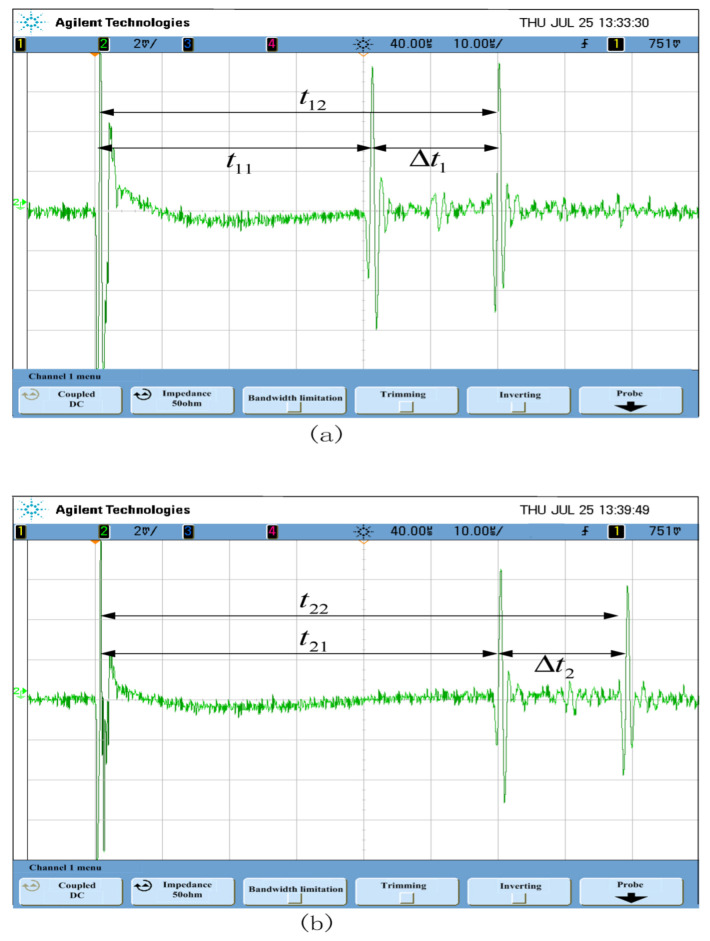
The magnetic sound signal collected from the experiment.

The signal detected by the magnetoacoustic signal receiver is shown in Figure 15. When the sampling radius is ${\;r}_{1}=75\;\mathrm{mm}$, the waveform acquired is Figure 15a. When the sampling radius is ${\;r}_{2}=105\;\mathrm{mm}$, the waveform acquired is Figure 15b. On the oscilloscope, the vertical axis represents the signal amplitude, each cell represents 2 mv, the horizontal axis represents time, and each grid represents 10 μs of time. The pulse indicated by the left arrow is a magnetic pulse generated by the coupling of the magnetic field through the space. The speed of propagation through the space is $c=3\times 10^{8}\;m/s$, which is much higher than the speed of sound. Therefore, this moment is taken as the signal reference point. Two peaks appear at the back, corresponding to the two edges of the ring. When a receiver is moved, the delay time between the two peaks varies, but the time interval between the two peaks remains the same. It can be concluded from Figure 15, the receiver of the magnetic acoustic signal received two magnetic acoustic signals. The first signal at the end near the receiver was strong, and the second signal at the end away from the receiver was weak. The amplitude of the second magnetoacoustic signal decreases because the magnetic sound signal scatters and diffracts as it travels.

When the sampling radius of the receiver is $r_{1}=75\;\mathrm{mm}$, the time for the first waveform on the oscilloscope in Figure 15a is about 40 μs; that is, the time for the first boundary of the model to propagate to the transducer; the time for the first waveform is about 60 μs, the delay time between the two waveforms is $\Delta t_{1}=20\;\mu s$, which corresponds to the propagation time of the magnetic acoustic signal between the two boundaries of the model. As the sampling radius of the receiver increases, the delay time of both waveforms increases. As shown in Figure 15b, the delay time corresponds to the increase of the sampling radius, and the delay time between the two signals remains $\Delta t_{2}=20\;\mu s$.

From the experimental results, it can be seen that the two peaks collected on the oscilloscope are the magnetic and acoustic signals generated by the electromagnetic excitation of the two boundaries of the steel ring. Under this experimental system, the model can generate the magnetoacoustic signal by electromagnetic excitation.

## 5. Conclusions

In this paper, a new method based on magnetoacoustic coupling imaging is studied. This method has great potential in rail defect detection. The electromagnetic field equation and the acoustic pressure wave equation of the steel ring model are deduced, and the simulation calculation and experiment are carried out.

(1) Firstly, the finite element method is used to carry out the simulation calculation. The relationship between each physical quantity in the rail is calculated under the pulse electromagnetic excitation. The simulation results are as follows: the relationship between the current density distribution in the rail and the excitation electromagnetic signal is obtained, the corresponding relationship between Lorentz force and the current density is calculated, and the motion characteristics of the particles in the steel ring under the action of Lorentz force are described. The results show that the Lorentz force properties in the model are influenced by the current density properties in the model and the static magnetic field. The Lorentz force influences the vibration characteristics of the solid particle. The current density corresponds to the conductivity distribution. In summary, the motion characteristics of the particles reflect the conductivity distribution of the steel ring.
(2) The 3D model was built, the waveforms of the magnetic acoustic signals are simulated. Next, the propagation of the original sound field is simulated. Finally, the influence of the position of the receiver on the intensity of the magnetic acoustic signal is studied.
(3) Through the simulation calculation of the steel ring model, it is found that the vibration velocity and displacement of the particle in the radial direction are much larger than those in the symmetrical direction. Therefore, in the detection of the magnetic acoustic signal, the direction of the magnetic acoustic signal receiver should be the same as that of the original sound field, and the detected sound signal is the strongest. This conclusion can provide theoretical guidance for the selection of the position of the receiver in the future detection.
(4) The results of simulation and experiment show that the magnetic acoustic signal can be generated by the steel ring under the excitation of electromagnetic signal, and the magnetic acoustic signal corresponds to the conductivity distribution in the model, so the conductivity distribution of steel ring can be reconstructed by the magnetic acoustic signal, the defect distribution of the steel ring can be detected.

The results of this paper can be applied to the study of rail micro-defect detection; because different detection techniques have their advantages and limitations, a single principle of detection technology will be unlikely to obtain all the information of rail state. The integration of multi-physical sensors, such as electromagnetic and acoustic sensors, is helpful to the quantitative assessment of rail defect damage and provide guidance for rail condition maintenance. The results may provide a new method for rail defect detection.

## Data Availability

Not applicable.
